# Myanmar mortality registration: an assessment for system improvement

**DOI:** 10.1186/s12963-017-0153-1

**Published:** 2017-09-25

**Authors:** Myitzu Tin Oung, Kerry Richter, Pramote Prasartkul, Viroj Tangcharoensathien

**Affiliations:** 1 Department of Medical Research, Pyin-Oo-Lwin Branch, Pyin-Oo-Lwin, Myanmar; 2 Institute for Population and Social Research, Mahidol University, Salaya, Nakhon Pathom, Thailand; 3 International Health Policy Program Foundation, Nonthaburi, Thailand

**Keywords:** Quality of mortality data, Vital registration, Myanmar

## Abstract

**Background:**

The vital registration system in Myanmar has a long history and geographical coverage is currently high. However, a recent assessment of vital registration systems of 148 countries showed poor performance of the death registration system in Myanmar, suggesting the need for improvement. This study assessed the quality of mortality data generated from the vital registration system with regard to mortality levels and patterns, quality of cause of death data, and completeness of death registration in order to identify areas for improvement.

**Methods:**

The study used registered deaths in 2013 from the vital registration system, data from the 2014 Myanmar Population and Housing Census, and mortality indicators and COD information for the country estimated by international organizations. The study applied the guidelines recommended by AbouZahr et al. 2010 to assess mortality levels and patterns and quality of cause of death data. The completeness of death registration was assessed by a simple calculation based on the estimated number of deaths.

**Results:**

Findings suggested that the completeness of death registration was critically low (less than 60%). The under-registration was more severe in rural areas, in states and regions with difficult transportation and poor accessibility to health centers and for infant and child deaths. The quality of cause of death information was poor, with possible over-reporting of non-communicable disease codes and a high proportion of ill-defined causes of death (22.3% of total deaths).

**Conclusion:**

The results indicated that the vital registration system in Myanmar does not produce reliable mortality statistics. In response to monitoring mortalities as mandated by the Sustainable Development Goals, a significant and sustained government commitment and investment in strengthening the vital registration system in Myanmar is recommended.

## Background

Reliable mortality statistics are invaluable for population health assessment, which contributes to evidence-based policy decisions, planning for health services, and effective interventions to address priority mortalities and monitor progress [1]. A well-functioning vital registration system (VRS) is universally recognized as the main source of mortality statistics because of its characteristics of universality, continuity, and permanence [2]. The VRS is able to generate mortality profiles of a population including cause of death (COD) information on a continuous basis at the national as well as the local level.

However, mortality statistics from the VRS are not available in many low- and middle-income countries [3]. Where the VRS is available, the data are often incomplete or unreliable, in particular COD information [1]. Only 14 out of the 75 low- and middle-income countries report mortality statistics based on death registration while only 81 countries out of 194 WHO member states report high- or medium-quality COD statistics [1]. In Africa and Asia, millions of people are born and die without being legally recorded in the country’s statistics [4] and only 1% of the population in Africa and Asia is currently living in countries which have a complete death registration system [5].

Myanmar does not have regular population censuses or surveys. After the 1983 Census, there were no censuses for three decades following. The most recent one was conducted in 2014, by the Department of Population (DOP) [6]. Hence, the VRS becomes the main source of mortality statistics of the country.

The registration of vital events in Myanmar began in 1904, in the lower part of the country, and gradually expanded into other areas until it was operational nationwide [7]. The current VRS was started in early 1962 in urban areas, and introduced in rural areas in 1979, in which the Central Statistical Organization (CSO) serves as the focal department and the Department of Public Health (DOPH) (formerly called the Department of Health) functions as an implementing organization [8, 9]. The responsibilities of CSO are to develop, provide, and distribute vital forms, to compile completed forms, to computerize and calculate vital statistics and to provide training and refresher training, supervision and monitoring on VRS. Registering vital events, data recording, issuing certificates and reporting are performed by health staff from the DOPH [8]. In 2001, the Myanmar-UNICEF Program 2001–2005 was implemented, which contributed to building a uniform VRS nationwide [9].

Currently, the VRS covers 321 towns in urban areas, and 287 townships in rural areas [10], with over 97% coverage. Nine townships, three from Sagaing Region and six from Shan State, cannot report vital information to CSO on a regular basis as they are geographically hard to reach.

Myanmar does not have a specific law for vital registration, and the nine related laws that are currently being applied [11] are not adequate nor effective to operate vital registration functions successfully. The most recent one is the Ward or Village Tract Administration Law (2012), which indicates that births and deaths must be informed to ward or village tract administrators, and the punishment for non-compliance would be the maximum seven days imprisonment or 5000 Myanmar Kyats (less than 5 US$) [12].

The regulation in the law is to inform births and deaths to local administrators, but deaths occurring in the community (around 80% of deaths) are recorded by midwives [11]. Due to the absence of a systematic notification process, deaths occurring in the community have to be collected primarily by midwives through household visits rather than being informed by the community. The COD information for community deaths was usually obtained from the family members, and its reliability is doubtful. For every death occurred in hospitals, the registered medical officer is responsible to record deaths and certify COD [11]. In the current situation, the responsible organizations cannot provide regular and/or refresher trainings on vital registration to every health staff from all states and regions to perform their functions efficiently.

Compared with births, registration of deaths is facing more challenges in reaching a satisfactory level of completeness and accuracy. The lack of effective laws to enforce mandatory registration, unequal access to services, no perceived benefits for registering deaths, low level of community awareness, and resource limitations are constraints on the efficiency and completeness of death registration in the country [13].

A recent VRS performance assessment of 148 countries using the Vital Statistics Performance Index (VSPI) shows a low VSPI score in Myanmar (0.02) compared with neighboring countries such as Malaysia and Thailand (0.75 and 0.57, respectively) [14]. The poor performance of the country’s VRS points to the need for a detailed assessment to set the agenda for improvements.

In response to the Sustainable Development Goals for monitoring maternal and child mortalities, premature mortalities due to non-communicable diseases (NCD), and road traffic accidents [15], this study assessed the quality of mortality data with regards to mortality level and pattern, quality of COD data, and completeness of death registration of the 2013 VRS. This detailed assessment is needed in order to identify areas for improvement and make actionable recommendations.

## Methods

### Data sources

The data and sources used in this study are the number of registered deaths by gender, age, and region from the 2013 VRS, the size of the population and reported deaths by gender, age, and region from the 2014 Myanmar Population and Housing Census, mortality indicators from published reports of the 2014 Census and mortality indicators, number of deaths by gender and age, and COD information of the country estimated by international organizations, such as the World Health Organization (WHO), the United Nations (UN) and the US Census Bureau, and from Global Burden of Disease Study 2015 (GBD 2015). The methodologies used for mortality estimation by international organizations can be reviewed at their websites [16–20].

### Assessment methods

The assessment of the quality of mortality data applies the systematic data quality assessment guide by AbouZahr et al. 2010, which verifies internal validity and coherence of mortality data from the VRS by comparing with data from other sources [21]. In applying the assessment guide, this study used independent sources of mortality data such as international organizations and the 2014 Myanmar Census. This guide is preferable in assessment of quality of mortality data as it describes relatively simple ways to assess the consistency and plausibility of mortality levels, patterns, and COD information. These simple checks can be applied as a routine practice to diagnose weaknesses and problems in mortality data at all levels. The information obtained can assist users and decision-makers in interpretation of quality of mortality data and for developing strategies to improve data quality [21].

The guide indicates how to execute basic calculations of mortality indicators, such as crude death rates, age and sex-specific death rates, ratio of male to female age-specific mortality rates, child mortality rates, percent distribution of deaths by age, and describes how to identify the distribution of major COD, age patterns of broad groups of COD, leading COD, ratio of NCD deaths to communicable disease (CD) deaths, and percentage of ill-defined COD. The method reviews the calculated mortality indicators and COD information and compares them with what would be expected for the given population estimated based on many decades of observations of demographic and epidemiological trends in different settings [21].

The completeness of death registration was assessed by a simple calculation (i.e., dividing the number of registered deaths by the total estimated number of deaths for the same period), and then multiplying by 100 [22]. The mortality estimates from the GBD 2015, the UN and the 2014 Myanmar Census were used as independent data sources to estimate the completeness of death registration. The reliability of the estimates based on this method depends on the reliability of the independent data source [22].

This method was also applied to estimate the completeness of death registration for each state or region of the country. The adjusted mortality indicators by region, such as Infant Mortality Rate (IMR), under-5 Mortality Rate (U5MR), and Crude Death Rate (CDR), estimated based on the data from the 2014 Census [23] were used to assess the completeness of death registration for each state or region. The differential completeness of the registration of deaths can provide baseline information about the performance of the VRS in each state and region.

In the 2014 Census, the infant and under-five mortality was measured from two questions for ever-married women aged 15 and over: the number of children ever born alive and the number of children died (or survived). The data were compiled into the proportion of children ever born who had died by the age of their mother, and these data were transformed into infant and child mortality rates using the indirect demographic method (the Brass method) [24, 25].

The census estimated adult mortality by applying the Brass Growth Balance method (BGB) [24, 25]. The estimation was based on the question on the number of deaths (by age and sex) in a household during the 12 months prior to the census [23]. The CDR at the national level was calculated based on adjusted child and adult mortality. Mortality rates for each state and region were calculated in the same way [23]. The census report said, according to the BGB method, the percentage of under-reporting of deaths was 29.7% for males and 37.5% for females at the national level [23]. However, given the poor performance of the BGB method, largely due to the strong likelihood that the assumptions which underlie the method are not replicable in populations such as Myanmar, these estimates of under-reporting of deaths in the census should be viewed extremely cautiously, and are likely to severely under-estimate the true extent of under-reporting of deaths.

### Preparation for the assessment of the quality of mortality data from the VRS

Before applying the method, the quality of age and sex structure of population from the census was assessed by constructing the age-sex pyramid of the population and calculating Whipple’s index, Myer’s index, age and sex ratios, and joint score index.

The age-sex pyramid of the population shows minor age misreporting, especially ages ending in 0 and 5. The Whipple’s index was 123. It means that quality of data is acceptable as the deviation from perfect is less than 25%. The Myer’s Blended index was 9.7 for male and 9.9 for females, indicating minor age misreporting, with preference for ages ending in 0 and 5. The age ratios of both male and female were close to 100 across age groups from 15 to 64 years. The sex ratio pattern followed the typical one (i.e., the sex ratios are slightly over 100 at the early ages due to more male than female births, and are reduced continuously, starting from age of 20 up to the oldest ages, since mortality is usually higher for males than females). The joint score (JS) index is 13 (JS < 20: accurate of age and sex structure) [26]. The findings suggest the age and sex structure of the census population was fairly accurate within the age ranging from 15 to 64 with observably fewer males than females in adulthood. Such pattern can be seen in a population where there is an excess adult male mortality, or where out-migration is male-dominated [26].

Then, to calculate mortality indicators based on the 2013 registered deaths, the mid-year population for 2013 was estimated from the numbers of population collected in the 2014 Census by applying the annual population growth rate of 0.89% [27].

## Results

In the 2013 VRS, there are a total of 199,491 registered deaths. Among all deaths, 82.9% took place at home and 24.2% were certified COD by registered medical doctors in attendance or not in attendance (16.9% and 7.3%, respectively).

### Assessment of levels of 2013 mortality in the VRS

#### Crude death rates

The crude death rate (CDR) calculated based on the 2013 VRS is 3.9 per 1000 population, about half that estimated for the country by other organizations: WHO (8.4) [28], UN (8.3) [29], US Census Bureau (8.0) [30], GBD 2015 (7.7) [31], and the 2014 Myanmar Population and Housing Census by the DOP (9.7) [23]. Demographers suggest that a CDR in developing countries below 5 per 1000 is not plausible, indicating under-registration of deaths [21].

A wide-ranging CDR is noted across fifteen states and regions, from 1.3 in Rakhine to 6.5 in Yangon. The urban CDR (6.1) is double that of the rural CDR (3.0). The evidences suggest wide disparities of incompleteness of death registration at the sub-national level and more severe under-registration of deaths in rural areas than urban areas. The male CDR (4.7) is higher than the female CDR (3.1). This gender difference in mortality can also be found in other populations.

#### Child mortality rates

The infant and under-five mortality at the national level estimated based on the 2013 VRS are 10.6 and 12.8 per 1000 live births respectively. This level is much lower than the estimates from the 2014 Census (IMR 62 and U5MR 72) [27] and other estimates: the UN (2010–1015) (IMR 46 and U5MR 60) [29] and the US Census Bureau (2013) (IMR 46 and U5MR 60) [30]. This indicates serious under-registration of infant and child deaths.

### Assessment of the patterns of 2013 mortality in the VRS

#### Age and sex-specific death rates

**Fig. 1:**
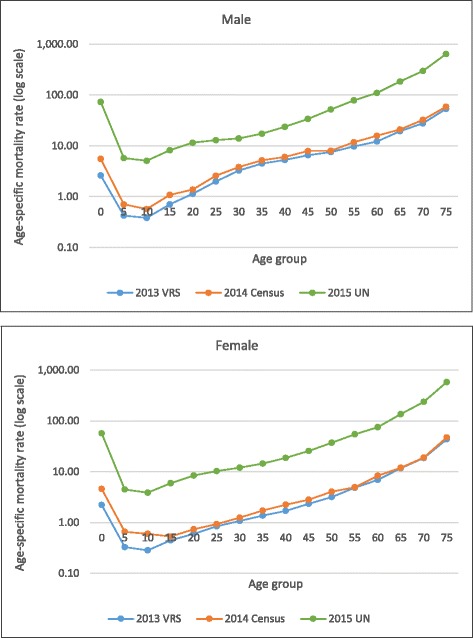
Logarithmic scale of age-specific mortality rates (male and female). Data source: registered deaths in the 2013 VRS, Central Statistical Organization; reported deaths in the 2014 Census (unadjusted), Department of Population; the United Nations, World Population Prospects: 2015 Revision (Estimates for Myanmar in 2010–2015)

The logarithmic scale of age-specific mortality rate (ASMR) from three data sources, namely the 2013 VRS, the 2014 Census (unadjusted), and the UN estimate [29] shows similar patterns a J-shaped curve emerges (i.e., higher mortality rates for the very young and the very old). The consistent pattern of ASMR across the three data sources in both male and female reflects that the 2013 VRS has minimal age and gender misreporting (Fig. 1).

In Fig. 1, it is noted that the level of ASMR by the UN estimate is obviously higher than that from the 2014 Census due to under-reporting of deaths in the census; and the 2014 Census ASMR levels are slightly higher than the 2013 VRS due to under-registration of deaths. A significantly lower level of under-five mortality in the VRS is noted compared with the UN estimate and the 2014 Census, reflecting severe under-registration of this age group.

#### Age distribution of deaths

**Fig. 2:**
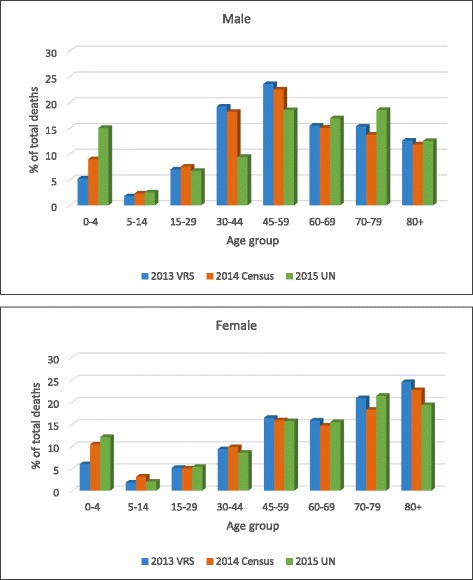
Age distribution of deaths (male and female). Data source: registered deaths in the 2013 VRS, Central Statistical Organization; reported deaths in the 2014 Census (unadjusted), Department of Population; the United Nations, World Population Prospects: 2015 Revision (Estimates for Myanmar in 2010–2015)

The percentage distribution of deaths by age registered in the 2013 VRS is significantly lower among under-five children, more obviously in male, when compared with the 2014 Census (unadjusted) and the UN [29] (Fig. 2), supporting the finding of severe under-registration of under-five children deaths. However, both the 2013 VRS and the 2014 Census produce higher male mortality among the middle age groups (30–44 and 45–59 age groups) compared with the UN estimates. The findings indicate a higher male mortality of the country in the middle age groups than the UN estimates for Myanmar.

#### Ratio of male to female mortality rates

In general, mortality rates tend to be higher in males than females at all ages, especially among young adults aged 15–35 years, because young males are more likely to die due to violence, road traffic accidents, and other external causes [21]. This condition produces higher male to female mortality ratios (greater than one) at all ages.

**Fig. 3:**
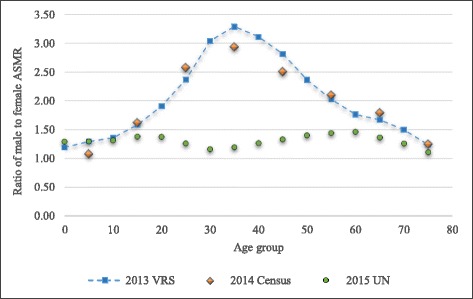
Male to female age-specific mortality ratio. Data source: registered deaths in the 2013 VRS, Central Statistical Organization; reported deaths in the 2014 Census (unadjusted), Department of Population; the United Nations, World Population Prospects: 2015 Revision (Estimates for Myanmar in 2010–2015)

While the ratios for male to female mortality rates in the 2013 VRS are greater than 1.0 at all ages, it is noted that they are very high in the middle age groups, reaching a peak of 3.3 in the 35–40 year group. This pattern is quite similar to the 2014 Census (unadjusted), where the male to female ratios are higher than 1.0 in all age groups, reaching a peak of 3.0 among middle age groups (Fig. 3).

However, this is different from the pattern of male to female mortality ratios calculated based on the number of deaths estimated by the UN [29]. In which, male excess mortality occurs among younger age groups and older adults aged 40–65 years but the ratios do not reach over 1.5. In contrast to the pattern produced by the VRS, less gender difference can be observed among age groups of 25–40 years (Fig. 3). The obvious difference in the middle age groups between two data sources, i.e., the 2013 VRS and the UN, might result from the possible under-estimation of male deaths in those age groups by the UN. However, this cannot be confirmed with the currently available information.

### Assessment of the quality of 2013 COD data in the VRS

#### Distribution of major COD

To verify the plausibility of COD data in the VRS, all COD are re-classified into three broad groups in line with the Global Burden of Disease groups. Group I consists of infectious and parasitic diseases, maternal causes and malnutrition. Group II includes non-communicable diseases and mental health conditions, and Group III includes injury-related deaths [21]. Comparing the 2013 VRS with the GBD 2015 estimates for Myanmar [31], it can be observed that the VRS has a higher percentage of deaths due to Group II COD and relatively lower percentages of deaths due to two other groups, particularly Group I COD (Table 1).

**Table 1:** Percentage distributions of three broad groups of diseases for COD

Broad diseases groups	2013 VRS^a^	GBD 2015^b^
Group I	13.4%	23.6%
Group II	79.8%	69.1%
Group III	6.8%	7.3%

Data source:

^a^Registered deaths from the 2013 VRS, Central Statistical Organization; ^b^Causes of death estimates for Myanmar in 2013 by GBD 2015

#### Age pattern of broad groups of COD

The age pattern of COD by three broad groups of diseases produced from the 2013 VRS is similar to the pattern estimated for Myanmar in 2013 by the GBD 2015 [31]. It is noted that deaths from Group I are most prevalent among the 0–4 age group and gradually decline with age. Deaths from Group II increase continuously with age, while deaths from Group III are prevalent among young adults, with a peak at the 15–29 year group in males, and the 5–14 year group in females.

**Fig. 4:**
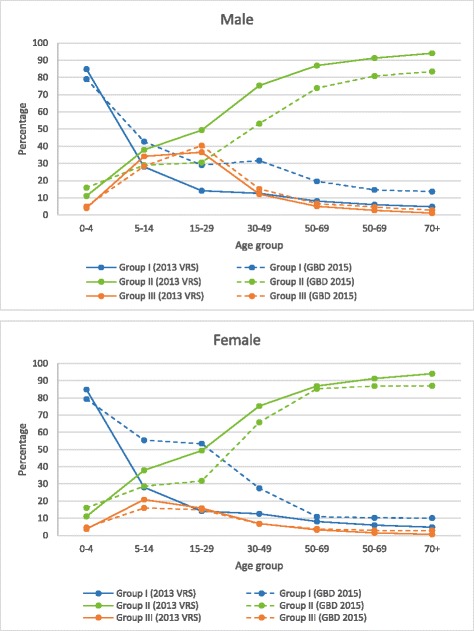
Percentage distributions of deaths by age group and major causes (male and female). Data source: registered deaths in the 2013 VRS, Central Statistical Organization; WHO, Global Health Estimate 2015 for Myanmar: Deaths by Cause, Age, Sex, by Country and by Region, 2000–2015

In the youngest age group, the percent of COD distributions by three broad groups of diseases is similar between two data sources in both male and female groups (Fig. 4). Apart from this age group, the percentages of Group II COD are higher and of Group I COD are lower in both males and females in the 2013 VRS. The differences are more significant in the middle age groups, especially Group I COD in females (Fig. 4). The percentages of Group III COD are more or less similar between two sources in all age groups.

#### Leading COD

Generally, in countries with an aging population and an increased life expectancy, the major COD are NCD. In populations with low life expectancy, most deaths are caused by infectious diseases and maternal conditions [21]. Based on the data from the 2013 VRS, in Myanmar with the life expectancy at birth of 64.7 years, tuberculosis, which ranks eighth, is the only infectious disease in top 10 leading causes of death (Table 2). The finding suggests that there are some problems in certification of cause of death or coding practices. According to findings from GBD 2015, tuberculosis and lower respiratory tract infection are third and fifth leading causes of death in 2015, respectively [31].

**Table 2:** Top 10 causes of death in the 2013 VRS

	ICD-10	Causes of death	Percentage
1.	I60–69	Cerebrovascular disease	13.1
2.	I10–15	Hypertensive disease	10.3
3.	I30–52	Heart disease (heart failure & complications of heart disease)	8.1
4.	K70–77	Disease of liver	5.0
5.	C15–26	Malignant neoplasm of digestive organs	3.7
6.	J40–47	Chronic lower respiratory diseases	3.5
7.	F10–19	Mental and behavioral disorder due to psychoactive substance use	3.2
8.	A15–19	Tuberculosis	2.2
9.	C30–39	Malignant neoplasm of respiratory and intrathoracic organ	2.1
10.	E10–14	Diabetes mellitus	1.6
Total			52.6

Data source: 2013 VRS, Central Statistical Organization

#### Ratio of NCD to CD in the COD

The ratio of NCD to CD is higher in countries with advanced epidemiological transition [21]: a ratio of 12.6 is noted among high-income countries; 8.1 among upper-middle-income countries; 1.8 among LMIC [32]. The ratio of NCD to CD for Myanmar in 2013 estimated by the GBD 2015 is 2.9 [31]. In Myanmar, a country from the LMIC group, the data from the 2013 VRS produced this ratio of 6.0, which was two times higher than the GBD 2015 estimate. All the above findings suggest a possible bias in assigning NCD codes to the COD.

#### Ill-defined COD

There are two sources of ill-defined (garbage) codes, including deaths classified as ill-defined (the R codes that describe symptoms and signs related to deaths), and deaths coded with vague or unspecific diagnoses such as cardiac arrest, hypertension, etc. [21]. The percentage of ill-defined COD estimated from the 2013 VRS is 22.3% of total deaths, where the R code accounted for 21.6%, reflecting the poor quality of COD certification.

### Assessment of completeness of 2013 death registration in the VRS

The completeness of death registration was 48.6% (95% CI: 34.7–70.5%) if calculated based on the estimated number of deaths from the GBD 2015 [31]; it was 46.9% when the estimated CDR from the UN (8.3 per 1000 population) was used [29]; and 40.3% when the estimated CDR from the 2014 Census (9.7 per 1000 population) was used [23].

In order to provide the baseline information about the performance of the VRS in each state and region, the completeness of death registration for each region was calculated based on the adjusted mortality indicators by region estimated from the 2014 Census [23] (Table 3). The completeness widely ranges from 14% in Rakhine to 66% in Yangon. In general, the regions located in the plains area, where people usually have better access to health and any other facilities (such as Yangon, Nay Pyi Taw, Mandalay, Magway, and Bago), have a higher level of completeness (above 45%). On the other hand, Rakhine, Shan, and Kachin States — where there is a long history of political instability and poor accessibility to health centers or registration units due to extreme difficulties in transportation — have the lowest level of completeness with 25% or less. The results show that several states and regions (10 out of 15) have less than 20% completeness of registration of infant and child deaths (Table 3).

**Table 3:** Completeness of death registration by residence, gender, and region (ordered by percent completeness of death registration for total deaths by region)

	2013 VRS^a^			2014 Census (Adjusted)^b^			Completeness of death registration (%) ^c^		
	CDR	IMR	U5MR	CDR	IMR	U5MR	Total deaths	Infant deaths	Under-5 deaths
UNION	3.9	10.6	12.8	9.7	61.8	71.8	40.3	17.2	17.8
Residence									
Urban	6.1	25.1	29.7	–	41.0	46.3	–	61.2	64.1
Rural	3.0	5.9	7.4	–	67.2	78.8	–	8.8	9.4
Gender									
Male	4.7	11.6	13.9	–	69.9	81.3	–	16.6	17.1
Female	3.1	9.6	11.7	–	53.6	62.0	–	17.9	18.9
Region									
Yangon	6.5	22.8	27.3	9.8	44.9	51.0	66.7	50.8	53.5
Nay Pyi Taw	3.7	15.7	17.9	7.2	55.4	63.9	51.7	28.3	28.0
Bago	4.7	8.7	10.8	9.8	61.9	72.0	48.5	14.1	15.0
Magway	5.6	15.3	18.1	11.6	83.9	100.6	48.2	18.2	18.0
Kayah	3.7	17.1	20.4	8.1	60.1	69.7	46.4	28.5	29.3
Mandalay	4.6	15	17.6	10.1	50.3	58.4	45.8	29.8	30.1
Mon	4.9	12.2	14.5	12.1	41.9	47.3	40.4	29.1	30.7
Ayeyarwady	3.1	7.3	9.2	9.9	86.2	103.6	31.4	8.5	8.9
Tanintharyi	2.7	4.5	6.5	8.6	70.8	83.4	31.0	6.4	7.8
Kayin	3.3	6.8	9	10.8	53.6	61.6	30.6	12.7	14.6
Chin	3.1	7.2	10.9	10.8	75.5	89.6	29.0	9.5	12.2
Sagaing	2.7	8.2	9.5	9.5	60.0	69.6	28.8	13.7	13.6
Kachin	2.2	3.9	5.4	8.8	52.8	60.6	25.4	7.4	8.9
Shan	2.0	5.8	7.4	8.4	55.5	64.0	24.0	10.5	11.6
Rakhine	1.3	4.4	5.5	9.7	61.1	71.0	13.6	7.2	7.7

Data source:

^a^Calculated based on registered deaths from the 2013 VRS, Central Statistical Organization

^b^Adjusted mortality indicators from Thematic Report on Mortality, Census Report Volume 4-B, the 2014 Myanmar Population and Housing Census, Department of Population

^c^Completeness of death registration was calculated by dividing the number of registered deaths by the total estimated number of deaths for the same period, and then multiplying by 100

**Fig. 5:**
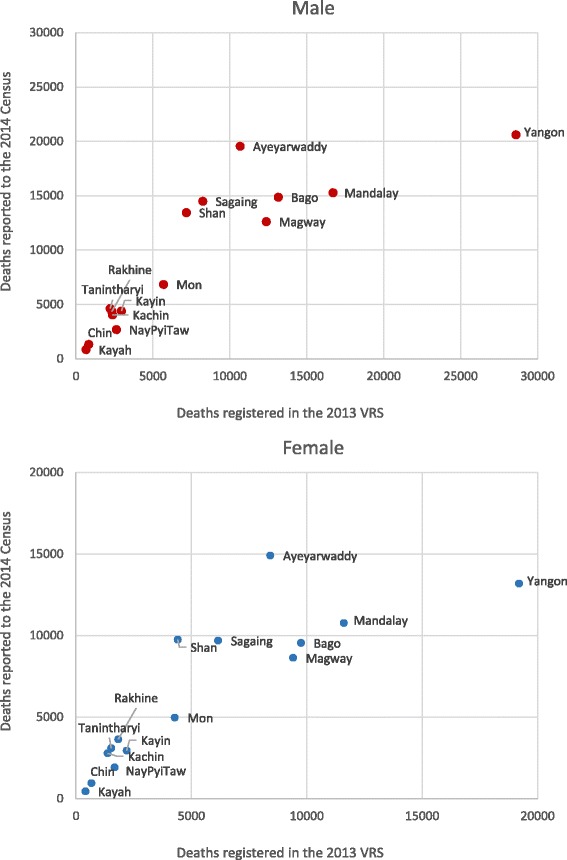
Comparing death counts from the 2013 VRS and the 2014 Census (male and female). Data source: registered deaths in the 2013 VRS, Central Statistical Organization; reported deaths (unadjusted) in the 2014 Census, Department of Population

Figure 5 in Appendix shows the discrepancy between registered death counts in the 2013 VRS and reported death counts (unadjusted) in the 2014 Census for each state and region. In the figure, the numbers of registered deaths were lower than the reported deaths to the census in many states and regions. On the other hand, compared with reported deaths to the census, higher number of registered deaths was observed in Yangon and Mandalay Region for male deaths, and in Yangon, Mandalay, and Magway Region for female deaths. This finding supported the results of relatively higher level completeness of deaths registration in those regions (around 50%, see Table 3), indicating that they have a better death registration practice compared to other areas. However, the degree of discrepancy between two data sources does not always reflect the level of completeness of death registration of the region because it also depends on the size of population of the area as well as the extent of under-reporting of deaths in the census.

## Discussion

The study assessed the quality of mortality data from the 2013 VRS in terms of level and pattern, quality of COD data, and completeness of death registration. The findings of implausibly lower levels of CDR at the national and sub-national levels and child mortality calculated based on the 2013 registered deaths suggest that a high proportion of deaths was not registered.

The results showed serious under-registration of infant and under-five children deaths in particular. This confirms studies from other countries. In China, the capture-recapture method reported that under- registration among children under-five was 21.6%, compared with the 13.0% overall under-registration rate [33]. In Thailand, the under-registration rate among children under-5 in 2005–2006 was very high (42.8%), even while death registration was almost universal (98.4% completeness in the Survey of Population Change) [21, 34]. In South Africa, only 42.0% of deaths of children under one year were registered in 2007 [35]. The findings highlight the need of special attention to develop effective strategies for improving registration of child deaths.

Lower death registration in rural areas compared to urban areas has been uncovered by this study, but it is not uncommon in developing countries having weak civil registration and vital statistics (CRVS) systems. A study in 32 countries reported that geographic dispersion and lack of transportation are factors hampering effective birth and death registration [36]. Observations in the VRS of Myanmar show that there is no requirement for registering deaths to obtain certificates for cremation or burial in rural areas. Improved regulations for burial certificates requiring death registration is one potential intervention to improve completeness, especially for deaths taking place in the communities.

Comparing age-specific mortality rates and age distribution of deaths among different data sources shows more or less similar pattern for all age groups. The results indicate that the registered deaths into the VRS do not have severe age and gender misreporting, suggesting that the registrars (i.e., basic health staff) are fairly reliable in terms of recording information of the deceased such as age and sex.

Age distribution of male deaths in the middle age groups was higher in the 2013 VRS and in the 2014 Census than the UN estimates. At the same time, very high male to female ASMR ratios were found in the middle age groups in the 2013 VRS and in the 2014 Census compared with the UN estimated deaths. These findings suggest that the country experienced a significantly higher male mortality in the middle age groups compared with females, which exceeds the international estimates.

The assumption of increased adult male mortality was made because of an increasing trend of injury-related deaths in the country since 2005 [37]. The death rate due to injury has approximately doubled during the 10-year period, from 2002 (2.4 per 100,000 population) to 2012 (4.4 per 100,000 population) [38]. Injury-related mortality was highest among those 15–44 year age groups and the proportion of deaths among males was four times higher than females in 2013 [39]. The high level of adult male mortality could also be caused by behavioral factors of unsafe and risky life styles among males, which seem to be deeply rooted in Myanmar socio-cultural context more than in other countries, such as tobacco and alcohol use, deficient self-health care habits, and negligence to disease symptoms [23]. This finding needs to be confirmed by conducting a survey or by comparing with other reliable data sources to take necessary actions for prevention of excessive premature male deaths.

The COD by three broad disease groups provided by the 2013 VRS are not consistent with the GBD 2015 estimates for Myanmar. The COD in the VRS is dominated by NCD, as reflected by the relatively higher proportion of Group II COD in all age groups apart from children under-five, the higher NCD to CD ratio, and higher contribution of NCD in the top ten COD. The high proportion of NCD dominating COD is a reflection of poor quality COD certification or coding.

In developing countries, most of the deaths take place outside health facilities and many deaths are not medically certified by qualified doctors; or, even if they are certified, the data are not reliable due to failure to meet the required standards [21]. A total of 82.9% of deaths in Myanmar, according to data from the 2013 VRS, and 65.0% of deaths in Thailand [40], took place at home, and the COD were not usually assigned by qualified medical doctors. Thus, in resource-poor settings, the quality of COD is always low due to the lack of institutional capacity, resources or in-service trainings for registrars in certification, and use of ICD-10 coding.

The proportion of ill-defined COD is used as an indicator for the assessment of quality of COD. When there is a high proportion of ill-defined COD, the COD distribution can be biased [21]. The result showed the ill-defined COD in the 2013 VRS in Myanmar was high (22.3% of total deaths). It is generally defined that the COD is of “low quality” if the proportion of ill-defined codes out of total deaths exceeds 20% [41]. Although the proportion of ill-defined COD was high, it is lower than other neighboring countries which have a better VRS. In Thailand, the proportion was 40.0% in 2008 [21] which had 98.4% coverage of death registration [34]; 46.0% of COD was ill-defined in Sri Lanka for 1950–1996 where death registration was 74% [41]. Despite Myanmar’s comparatively lower level of ill-defined COD in relation to neighboring countries, a very high proportion of non-ill-defined COD were certified by non-physician health workers (75.7% of total deaths). This suggests the interpretation of COD must be done with care. One way to verify is the application of the verbal autopsy method for community deaths.

The completeness of death registration calculated based on the estimated number of deaths from different data sources ranges from 40 to 50% at the national level. The under-registration was found to be more severe in hard-to-reach areas with poorer access to communities by health care providers, who are playing a primary role in data collection of vital events, such as Rakhine (13.6%), Shan (24.0%), Kachin (25.4%), Sagaing (28.8%), and Chin (29.0%). The findings indicate that differences in socio-economic development, level of poverty, political instability, and inequitable access to health centers can influence the geographical variations in completeness of death registration across states and regions.

It is important to note that one of the data sources to assess the completeness of death registration at the national and sub-national level was the 2014 Census. As described in the methodology section, the census mortality indicators were estimated by using the Brass method for both national and sub-national level. In application of the BGB method to estimate adult mortality, the major limitations are the assumptions of population stability and closed to migration. Obviously, the study population can hardly meet those assumptions due to sizable emigration from Myanmar to neighboring countries and due to declining population growth rate [29], which can lead to a bias in the estimation. More importantly, although the assumption of closed to migration is usually relaxed to some extent at the national level, the issue becomes a greater concern at sub-national level, where a substantial proportion of the population has migrated [24, 25]. The important message here is that it is necessary to perform a careful interpretation of the results obtained from those data. To be able to provide a more accurate estimates of completeness at both national and sub-national level, a direct matching technique, known as a dual records system, should be applied [2].

## Conclusion and recommendations

Our assessment of the mortality data produced from the 2013 VRS indicates that the system could not produce reliable mortality indicators. Deaths are severely under-registered and the situation is worse in rural areas, in areas with difficult access to health services and child deaths. According to the findings, in Myanmar, where most deaths occur outside health facilities and only a quarter of deaths are certified by medical doctors, the quality of COD is poor, hence less useful for planning purposes. To identify barriers to generating better quality mortality statistics from the VRS, it is recommended to undertake a detailed evaluation of the VRS of the country using the WHO/UQ (World Health Organization/University of Queensland) comprehensive assessment tool [42] or the vital registration assessment framework by Chalapati Rao [3].

Any government has obligations, as committed to UN Sustainable Development Goals [15], to strengthen birth and death registration, as demanded by a number of mortality- and birth-related SDG indicators (Table 4). SDG also mandates countries to achieve reduction in several cause-specific mortalities, for which the quality of COD is essential. For improving the VRS in Myanmar including the quality of COD, a significant and sustained commitment from the government is urgently needed.

**Table 4:** Sustainable Development Goals: Indicators related to CRVS [15]

Indicator 3.1.1: Maternal mortality ratio Indicator 3.2.1: Under-5 mortality rate Indicator 3.2.2: Neonatal mortality rate Indicator 3.4.1: Mortality rate attributed to cardiovascular disease, cancer, diabetes, or chronic respiratory disease Indicator 3.4.2: Suicide mortality rate Indicator 3.6.1: Death rate due to road traffic injuries Indicator 3.7.2: Adolescent birth rate (aged 10–14 years; aged 15–19 years) per 1000 women in that age group Indicator 3.9.1: Mortality rate attributed to household and ambient air pollution Indicator 3.9.2: Mortality rate attributed to unsafe water, unsafe sanitation and lack of hygiene (exposure to unsafe Water, Sanitation, and Hygiene for All (WASH) services) Indicator 3.9.3: Mortality rate attributed to unintentional poisoning Indicator 1.5.1, 11.5.1 and 13.1.2: Number of deaths, missing persons and persons affected by disaster per 100,000 people Indicator 16.1.1: Number of victims of intentional homicide per 100,000 population, by sex and age Indicator 16.9.1 Proportion of children under 5 years of age whose births have been registered with a civil authority disaggregated by age Indicator 17.19.2: Proportion of countries that (a) have conducted at least one population and housing census in the last 10 years; and (b) have achieved 100% birth registration and 80% death registration	

To improve the country’s population health by strengthening VRS, Myanmar has joined the Data for Health Initiative program in 2016. The program includes six interventions: strengthening vital registration practice and transmission procedures; implementation of automated verbal autopsy for community deaths; improving the quality of medical certification of COD for hospital deaths; strengthening International Classification of Diseases (ICD) coding systems; providing training in estimation of completeness and quality of birth and death registration; and improving capacity to assess and monitor quality of vital statistics data.

Under the Bloomberg Data for Health Initiative, two of the above interventions, the introduction of the verbal autopsy process to identify COD for community deaths as well as providing trainings on certification of COD to medical doctors, have already been initiated in some townships of Sagaing, Magway, and Mon to improve the quality of COD. The application of the process needs to be expanded nationwide to be able to generate reliable COD data at both local and national level so that the data can translate to public health policy and interventions.

In addition, social mobilization to increase public awareness, which is the center of success for completeness of death registration, should also be implemented in conjunction with legislation and enforcement of a vital registration law, which are legal platforms supporting the completeness of vital registration [2]. The requirement of a death certificate as a condition to gain a burial or cremation approval, and the development of an effective notification system, which ensures all deaths are reported to the health staff by the community, are possible actions to increase the completeness of death registration.

## Abbreviations

ASMR: Age-specific Mortality Rate
CD: Communicable Diseases
CDR: Crude Death Rate
COD: Cause of Death
CRVS: Civil Registration and Vital Statistics System
CSO: Central Statistical Organization
DOP: Department of Population
DOPH: Department of Public Health
ICD-10: International Classification of Diseases, 10th revision
IMR: Infant Mortality Rate
LMIC: Lower- and middle-income countries
NCD: Non-communicable Diseases
SDG: Sustainable Development Goals
U5MR: Under-5 Mortality Rate
UN: United Nations
UNICEF: United Nations International Children’s Emergency Fund
VRS: Vital Registration System
VSPI: Vital Statistics Performance Index
WHO: World Health Organization
